# Frailty and Oncology: An Increasingly Common Combination

**DOI:** 10.1002/cam4.71499

**Published:** 2025-12-26

**Authors:** Yanira Hernández‐Aguiar, Ángel Becerra‐Bolaños, Aurelio Rodríguez‐Pérez

**Affiliations:** ^1^ Department of Anesthesiology, Intensive Care and Pain Medicine Hospital Universitario de Gran Canaria Doctor Negrín Las Palmas de Gran Canaria Spain; ^2^ Department of Medical and Surgical Sciences Universidad de Las Palmas de Gran Canaria Las Palmas de Gran Canaria Spain

**Keywords:** frailty, palliative care, prehabilitation, prognosis, screening, therapeutic optimization

## Abstract

**Background:**

Frailty is defined by a reduction in physiological reserve and an increased vulnerability to stressors. In oncology, frailty is highly prevalent and has been consistently associated with a worse prognosis. The aim of this manuscript is to understand the interaction between frailty and cancer to optimise therapeutic decision‐making and improve patient‐centred outcomes.

**Methods:**

A narrative literature review was conducted using the PubMed database, with articles published up to July 2025 included. The search terms used included "frailty", "oncology", "cancer", "malignancy", "diagnosis", "optimisation", "treatment" and "prognosis". In accordance with the protocol, the following documents were prioritised: clinical guidelines, systematic and narrative reviews, observational studies, and randomised clinical trials.

**Results:**

Frailty has been shown to independently predict postoperative morbidity, chemotherapy toxicity, functional decline, and mortality. This can result in both undertreatment and overtreatment. Consequently, frailty assessment has emerged as a cornerstone of personalised oncology, enabling treatment individualisation beyond tumor characteristics alone. While a Comprehensive Geriatric Assessment remains the gold standard for frailty evaluation, screening tools should be used to facilitate risk stratification in routine practice. Incorporating frailty into decision‐making processes has been shown to reduce inappropriate undertreatment and overtreatment, improve treatment tolerance, and facilitate shared decision‐making. Multimodal, patient‐centred interventions, such as exercise, nutritional support, medication optimisation, psychosocial care, and early palliative integration, mitigate frailty, enhance quality of life, and support adherence to individualised therapeutic plans. Oncogeriatric models of care further operationalise personalised medicine by coordinating these interventions within multidisciplinary teams.

**Conclusion:**

It is crucial to acknowledge frailty as a pivotal clinical variable, rather than considering it a contraindication to cancer treatment. Health systems should promote structured frailty evaluation, professional training, and institutional pathways to ensure equitable, patient‐centred management of frail individuals with cancer. Integrating frailty into oncology clinical practice operationalises personalised medicine by shifting the focus from treating the disease to treating the whole patient.

## Graphical abstract

Frailty is an essential clinical parameter that should guide the individualization of therapeutic strategies in oncology to promote more effective, patient‐centered medicine. This narrative review explores the importance of incorporating frailty assessment and optimizing patients' clinical status to improve cancer treatment outcomes.

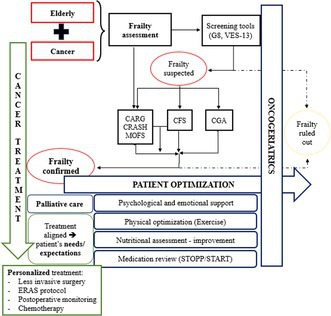

## 1 Introduction

Frailty is defined as a recognizable clinical state of increased vulnerability resulting from a decline in the physiological reserves and functions of multiple systems in the body. Frail patients have a reduced ability to adapt to disturbing factors such as acute illness, injury, or stress [1]. Frailty is particularly relevant in cancer patients since they tend to be older adults with multiple comorbidities, polypharmacy, and compromised functional status [2].

The presence of frailty negatively affects the clinical outcomes and quality of life of cancer patients, as it increases the rate of side effects from chemotherapy, postoperative complications, and mortality [3]. Frailty can also influence the selection of patients who are eligible for certain treatments [4]. Optimizing cancer patients prior to surgery or chemotherapy can reduce frailty, improving prognosis after treatment [5].

This narrative review examines the relationship between frailty and cancer from pathophysiological, clinical, and therapeutic perspectives. It examines the prognostic influence of frailty on cancer management and suggests strategies to optimize care for frail patients. The goal is to create more personalized medicine based on each patient's needs. To conduct this review, a literature search was performed in the PUBMED database for articles published in English up to July 2025. The following terms were used to conduct the search: “frailty,” “oncology,” “malignancy,” “cancer,” “diagnosis,” “optimization,” “treatment,” and “prognosis.” The review primarily includes clinical guidelines, systematic and narrative reviews, observational studies, and randomized clinical trials. The scale for the assessment of narrative review articles (SANRA) was used to guide this review [6].

## 2 Physiology of Aging and Frailty

Frailty is a condition closely linked to aging. However, not all older adults are frail. Therefore, it is important to understand the pathophysiological mechanisms linking frailty to aging:

- Musculoskeletal changes: sarcopenia, defined as the progressive loss of muscle mass, strength, and physical performance, is a fundamental component of frailty. This condition is influenced by various factors, including mitochondrial dysfunction, ongoing inflammation (a phenomenon termed “inflammaging”), hormonal deficiencies (particularly in testosterone and IGF‐1), insulin resistance, and decreased physical activity. Sarcopenia is associated with worse outcomes in cancer patients, such as lower tolerance to chemotherapy, greater toxicity, and reduced survival [7, 8, 9].
- Immunosenescence and immunological alterations: immunological aging is characterized by a decrease in adaptive immunity and an increase in basal systemic inflammation. This creates a double vulnerability, resulting in an increased risk of infection and decreased immune surveillance against tumor cells. This dysfunction is further exacerbated by cancer and immunomodulatory treatments [10, 11].
- Cardiovascular, respiratory, and neuroendocrine changes: arterial stiffness, ventricular hypertrophy, and endothelial dysfunction lead to reduced hemodynamic reserve. There is also a bidirectional relationship between cardiovascular disease and frailty, resulting in a vicious cycle. At the pulmonary level, decreased elasticity, forced expiratory volume, and diffusion capacity affect the body's response to exertion and increase surgical risk. Dysfunction of the hypothalamic–pituitary–adrenal axis and resistance to anabolic hormones directly influence metabolism and functional status. Metabolic syndrome and sarcopenia are interrelated through insulin resistance, adipose tissue, and vitamin D deficiency [12, 13, 14, 15].
- Neurological and cognitive changes: brain aging can impair executive function, processing speed, and motor coordination. Mild cognitive impairment and dementia are more frequent in frail patients and are associated with poorer treatment adherence, greater dependence, and increased complications. The concept of “cognitive frailty” has been proposed as a complementary entity to physical frailty, with the understanding that cognitive frailty is not to be confused with cognitive ability [16, 17].

In addition to the characteristics associated with aging, frailty can be induced or accelerated by the presence of a malignant tumor. Cachexia‐anorexia syndrome, tumor inflammation, functional obstruction, and the toxicity of cancer treatments can trigger or worsen a state of frailty [18, 19]. Similarly, frailty and related pathophysiological conditions become more significant in the context of active cancer [20]. It is essential to understand these mechanisms to effectively address frailty in a preventive and comprehensive manner. Conversely, immunological, epigenetic, and metabolic dysfunctions in a patient with a compromised immune system could lead to a more rapid development of cancer.

## 3 Assessment of Frailty in Cancer Patients

There are two widely accepted models for assessing frailty: the phenotypic model and the deficit accumulation index. The phenotypic model identifies five clinical criteria (weight loss, weakness, slowness, exhaustion, and low physical activity), and the deficit accumulation index quantifies comorbidities, functional impairments, and geriatric syndromes to generate a continuous index [21]. Both models have demonstrated predictive value in cancer patients. Systematic frailty assessment in cancer patients has emerged as a key strategy in personalized medicine for older adults. The following tools have proven useful in assessing frailty in cancer patients:

- Comprehensive Geriatric Assessment (CGA): considered the gold standard, the CGA covers multiple domains, including comorbidities, functionality (basic and instrumental activities of daily living), cognition, nutritional status, polypharmacy, emotional state, and social situation. The CGA can predict the onset of postoperative complications, oncological toxicity, and short‐ and long‐term mortality, as well as changes in the therapeutic plan [22, 23, 24].
- Screening Instruments: G8 and Vulnerable Elderly Survey (VES‐13). Since it may be impractical to apply a CGA to all patients in routine clinical practice, other tools are used to determine which patients would benefit from a more comprehensive assessment [25, 26]:
  ○ G8: this is a quick, validated tool consisting of eight items focused on nutritional status, mobility, polypharmacy, cognition, and age. A score of 14 or lower suggests a risk of frailty and the need for a CGA.
  ○ VES‐13: the 13‐item scale includes age, self‐perceived health, and functional limitations. A score of 3 or higher indicates vulnerability. The scale has been validated in geriatric and oncology settings.
- Clinical Frailty Scale (CFS): this visual and functional tool classifies patients into one of nine levels, ranging from “very fit” to “very frail.” It is useful in settings with limited clinical time, is based on clinical judgment, and is easy to apply. The scale has been validated for predicting adverse outcomes after surgery, chemotherapy treatment, and hospitalization for cancer patients [27, 28].
- Edmonton Frailty Scale: This scale has been shown to reliably identify frailty in surgical patients, even when administered by staff without formal geriatric training, through the assessment of various factors: polypharmacy, sphincter continence, cognition, general health, self‐reported health, functional independence, performance, social support, and mood [21].
- Modified Frailty Indices (mFI‐11, mFI‐5, and mFI‐6): despite being concise and quick to apply, mFI‐11 and mFI‐5 are able to predict the occurrence of adverse postoperative outcomes in all surgical specialties [21]. For patients undergoing cancer surgery, the mFI‐5 has been shown to be the most effective predictor of adverse events [29]. So, as a significant proportion of cancer patients undergo surgery, these user‐friendly scales could be highly beneficial for this population. mFI‐6 adds serum albumin to the traditional mFI‐5. For patients undergoing chemotherapy, the mFI‐6 has been shown to be useful in predicting who will be able to complete their treatment, anticipating serious adverse events and identifying those who could benefit from preventive interventions before treatment begins [30]. This confirms the link between frailty, nutritional status, and therapeutic adherence.
- Specific scales used for cancer patients:
  ○ Cancer and Aging Research Group Score (CARG): this tool integrates clinical, functional, and sociodemographic variables to predict chemotherapy toxicity in patients over 65 years. It has been widely validated and is useful in outpatient settings [31].
  ○ Chemotherapy Risk Assessment Scale for High‐Age Patients (CRASH): this scale combines clinical, functional, and laboratory factors. It estimates the risk of hematological and non‐hematological toxicity. Although it is time‐consuming and requires resources, it offers high prognostic accuracy [32].
  ○ Multidimensional Oncological Frailty Scale (MOFS): this innovative tool is designed to assess multiple dimensions relevant to oncogeriatrics, including physical function, sarcopenia, comorbidities, and dependency. Recent studies have shown that it correlates better with overall survival than the G8 or VES‐13 [33].

The incorporation of these evaluation instruments into the oncology treatment plan facilitates the prediction and mitigation of adverse effects and hospital readmissions, enhances adherence to treatment regimens, facilitates the organization of preventive measures (e.g., nutrition, exercise, psychology), fosters patient autonomy and shared decision‐making, optimizes healthcare resources, and ensures a more personalized management [34, 35]. The selection of the appropriate tool depends on the clinical objective (screening vs. diagnosis), the time available, and institutional resources. It is generally recommended that, in the event of a detected vulnerability, a screening instrument (G8 or VES‐13) be applied, followed by CGA. To determine the most effective systemic treatments, it is recommended to utilize CARG or CRASH in conjunction with functional scales such as CFS.

Clinical tools can be supplemented with epigenetic and metabolomic biomarkers, as they capture different aspects of biological aging [36]. Plasma proteomic signatures have been developed as frailty indices. A proteomic analysis of older adults revealed multiple plasma proteins associated with inflammation, lipid metabolism, and cellular senescence. These proteins were associated with existing frailty and the future risk of developing it [37]. Frailty indices have been developed using blood biomarkers, either as standalone tools or alongside clinical measures. The Canadian Longitudinal Study on Aging developed a 23‐item frailty index (FI‐Blood) based on blood‐based biomarkers, which has been shown to have independent prognostic value for mortality [38]. Other predictive models use combinations of laboratory markers such as C‐reactive protein, hemoglobin, albumin, 25‐hydroxyvitamin D, and free testosterone. These models have been shown to consistently predict frailty status and adverse events [39]. Biomarker‐based indices reflect the multi‐systemic nature of frailty, may improve risk stratification and monitoring, and reveal potential biological pathways for prevention.

## 4 Impact of Frailty on Cancer Prognosis

Frailty affects the administration of treatments. Among older women with breast cancer, frailty is associated with a lower likelihood of receiving adjuvant radiotherapy, chemotherapy, or surgical intervention. This leads to a significant decrease in survival. Furthermore, among frail patients who undergo surgery, they are more likely to undergo a mastectomy than more conservative surgery [40, 41]. A study of patients over 70 years of age with solid tumors (in which colorectal cancer was the most common diagnosis, accounting for 39% of cases) found an association between increased frailty markers and recommendations for “palliative treatments,” while the absence of frailty markers was linked to standard treatments [42]. Consequently, while age should not constitute a contraindication for radical rectal restorative surgery, frailty and functional capacity of patients should be judiciously evaluated when formulating a major surgical plan for rectal cancer [43], due to the increased risk of postoperative complications associated with frailty. For low‐risk, localized prostate cancer, frailty can guide the decision to pursue active surveillance or hormonal therapy instead of radical surgery or radiotherapy. Thus, the International Society of Geriatric Oncology (SIOG) recommends comprehensive functional assessments to tailor the therapeutic approach to each patient [44]. Due to the high morbidity and mortality associated with radical cystectomy, the SIOG also proposes evaluating alternatives such as transurethral resection or hypofractionated chemotherapy in frail patients with bladder cancer [25]. Although few studies have adjusted for patient frailty in colorectal or lung cancer surgeries, age has been associated with a lower likelihood of receiving treatment and lower survival rates compared to younger patients [45].

Frailty is an independent risk factor associated with higher postoperative mortality rates, increased complications, longer hospital stays, and the need for post‐discharge care [46]. However, many factors contribute to undertreating cancer patients, such as chronological age, physicians' beliefs, age bias, and the presence of comorbidities [45]. A panel of experts agreed that it is unethical to recommend treatment without first assessing the patient's frailty and giving them the opportunity to share their values, goals, and preferences. Although the panel failed to reach a consensus on the relationship between justice as a bioethical principle and overtreatment or undertreatment, they concluded that justice was related to undertreatment when an oncologist denied treatment solely based on the patient's age [47]. This emphasizes the importance of including frailty in the assessment of elderly cancer patients to determine the most appropriate treatment that aligns with their needs and expectations.

Cancer treatment is associated with frailty prevalence rates of up to 35%. Various factors, including advanced tumor stage, frequent chemotherapy cycles, anemia, leukopenia, comorbidities, and malnutrition, increase the risk of developing frailty during treatment [48]. Besides, the degree of frailty in frail patients may worsen during treatment, with partial recovery in some cases and persistence in others, which increases mortality and decreases quality of life [49]. Thus, although frailty is typically associated with age, it can also affect younger cancer patients. Both cancer and its treatments can reduce physiological reserves and make patients more vulnerable to stressors, regardless of their age. Rates of frailty and muscle mass decline in young adult cancer survivors have been found to be comparable to those of much older populations without cancer, reflecting accelerated aging and impaired resilience attributable to cancer and its treatment [50, 51].

## 5 Cancer Treatment in Frail Patients

The treatment of frail cancer patients requires a person‐centered, multidisciplinary approach tailored to their functional abilities, preferences, and life expectancy. According to international guidelines, CGA should be incorporated to evaluate the benefits and risks of treatment and to prevent or minimize complications resulting from cancer treatment [52]. Surgery is the main treatment for many solid tumors, but it can lead to higher morbidity and mortality in frail patients [53]. Less invasive techniques, such as laparoscopy and robot‐assisted surgery, and Enhanced Recovery After Surgery (ERAS) protocols can reduce postoperative complications and speed up patient recovery [54, 55]. In addition, postoperative monitoring of these patients should be increased to detect early signs of potential complications. This would allow for early treatment and prevent the complication from worsening and affecting the patient's postoperative evolution.

Frail patients are at greater risk of experiencing secondary chemotherapy toxicity (CTT), which, if not managed correctly, can lead to undertreatment of cancer. The most common CTT events are gastrointestinal, lymphatic/hematological, and skin‐related [56]. Most older patients can benefit from cancer treatment to the same extent as younger patients. Only a small percentage of patients should be excluded due to reduced tolerance [57]. Although frail patients may have a poorer quality of life initially, they experience less decline in overall, physical, and emotional quality of life after chemotherapy than non‐frail patients do. This is likely because frail patients have lower expectations about their quality of life during treatment [58].

Other treatments, such as hormone therapy for breast and prostate cancer, are generally well tolerated and may be an effective alternative for frail patients who are not candidates for chemotherapy or surgery. However, it has been suggested that androgen deprivation therapy is more likely to cause significant morbidity or even death in frail patients. Therefore, active surveillance is recommended to inform therapeutic decisions and compare the risks of worsening frailty with the risks of cancer progression [59]. Immunotherapy has also demonstrated efficacy in treating multiple tumors, including those of the lung, kidney, and melanoma. However, further evidence is needed regarding its use in frail patients due to the risk of immune‐mediated toxicities [60]. In cases of advanced tumors, immunotherapy can also be used as a type of palliative treatment. It has been shown to improve quality of life for patients, control their symptoms effectively, and reduce the risk of deterioration caused by the side effects of chemotherapy [61].

Even at extreme doses, radiotherapy may not worsen frailty and may have the potential to reduce it in suitable patients. Therefore, it can be safely administered to these patients [62]. Additionally, a combination of immunotherapy and intensity‐modulated image‐guided radiotherapy, or other radiotherapy techniques, may be beneficial for patients with locally advanced cancer who are not candidates for chemotherapy [63]. Palliative radiotherapy plays a crucial role in controlling symptoms and improving quality of life.

Palliative care should be integrated early on for patients with advanced frailty. This includes symptom management, emotional support, and decision‐making centered on life goals. Providing palliative care early on improves quality of life, reduces hospitalizations and expenses, avoids unnecessary interventions, and helps focus treatment on the patient's goals. This approach has the potential to enhance clinical outcomes while reducing costs [64]. Thus, the therapeutic approach for frail cancer patients should be guided by an extensive evaluation of risks and adaptation of treatments, while always considering the patient's preferences. Shared decision‐making and teamwork are key to providing humane and effective cancer care.

## 6 Useful Interventions for Frail Cancer Patients

Frailty is a dynamic and malleable process. Interventions have been shown to affect its assessment and are useful for all patients [65]. Therefore, especially in cancer patients, strategies aimed at improving functional reserve in frail individuals must be implemented alongside cancer treatment. However, cancer therapies should never be delayed. These interventions cover areas such as physical exercise, nutrition, pharmacological optimization, psychosocial support, and geriatric rehabilitation:

- Exercise programs offer several clinically relevant benefits and have positive effects on health‐related quality of life, physical functioning, social functioning, and fatigue [66]. They also have a variety of side effects related to cancer treatment and physical, functional, and psychosocial outcomes. These results are more pronounced with moderate‐ to vigorous‐intensity exercise programs than with low‐intensity programs [67]. Both aerobic and resistance exercises positively affect self‐esteem, physical fitness, body composition, and the ability to complete chemotherapy with no significant adverse effects [68]. However, the safety, feasibility, and benefits of exercise depend on the type of cancer and the desired outcome. Since there is no one‐size‐fits‐all approach, the prescription must be tailored to each patient and their specific cancer [66]. Home‐based programs are convenient and easy to follow. They require few resources and offer perceived health benefits and a sense of control over one's health [69].
- Nutritional status is a determining factor in the progression of frailty. Nutritional intervention must be initiated early and be customized to the patient. It should focus on increasing nutritional intake and reducing inflammation and hypermetabolic stress [70]. Interventions should include a nutritional assessment using one of the following scales: the Malnutrition Screening Tool (MST), the Malnutrition Universal Screening Tool (MUST), the Malnutrition Screening Tool for Cancer Patients (MSTC), or the Patient‐Generated Subjective Global Assessment Short Form (PG‐SGA SF). Screening can be avoided for individuals with a diagnosis or treatment plan carrying a high risk of malnutrition [71]. In these cases, nutritional treatment should begin immediately. Similarly, stimulating appetite and managing symptoms that interfere with food intake, such as nausea, mucositis, and dysgeusia, with the help of dietitians, is essential for the success of nutritional intervention. Combining exercise and nutrition enhances anabolic effects.
- Polypharmacy is common in older adults with cancer and contributes significantly to frailty. Drug interactions can occur between medications used to treat comorbidities, as well as between these medications and those used for chemotherapy or supportive care, particularly among those metabolized by cytochrome P450 isoenzymes [72]. Medication reviews help identify potentially inappropriate drugs, interactions, and duplications. This improves the quality of patient care and quality of life. It is recommended that predictive or drug‐specific algorithms and criteria, such as the Beers Criteria or the Screening Tool of Older Persons' Potentially Inappropriate Prescriptions (STOPP)/Screening Tool to Alert Doctors to Right Treatments (START), be applied. It is also recommended that questionnaires and guidelines be used, and that renal and hepatic function data be taken into account [73].
- Emotional and cognitive spheres must also be addressed. Psychological symptoms associated with cancer include anxiety, depression, fear of recurrence, and cognitive impairment. These symptoms tend to appear in clusters, which can make them difficult to identify [74]. The presence of anxiety and depression can directly impact both treatment adherence and cancer prognosis [75]. Frailty is significantly associated with an elevated risk of depression. Additionally, depression is a susceptibility factor for frailty [76]. For patients with breast cancer, interventions focused on physical or psychosocial factors, such as physical exercise, yoga, psychotherapy, or meditation, can improve depressive symptoms [77]. Additionally, rehabilitation improves the quality of life for cancer patients by improving functioning and reducing psychological distress [78]. Providing emotional support from the beginning improves patient resilience and encourages shared decision‐making.

Given the various interventions recommended for cancer patients, especially for frail patients, the implementation of oncogeriatric units has demonstrated consistent benefits in clinical practice, reducing therapeutic failure [79]. These units enable the assessment of frailty in cancer patients and the coordination of simultaneous interventions, such as nutrition, rehabilitation, and psychological support. They also allow for the adjustment of treatment and the redefinition of therapeutic objectives according to the patient's progress. Additionally, they stimulate specific research in oncogeriatrics, promote the training of healthcare professionals, and encourage information sharing. All of these actions are taken while considering the balance between risks and benefits and prioritizing patient expectations [80, 81]. Therefore, a geriatric assessment and multidisciplinary interventions could decrease the associated costs of cancer treatment by reducing complications and readmissions [82].

Despite the available evidence, the current rate of frailty assessments among cancer patients in clinical settings remains low. Only 52% of healthcare professionals assess frailty in all or most of their cancer patients, and just 32% use multidimensional instruments [83]. However, there are structural barriers to implementing this approach in oncology practice, such as a lack of geriatric oncology training, a lack of knowledge about the existence of specific tools, time constraints during consulting hours, and a lack of follow‐up services, resources, and investment [83, 84]. The solution requires implementing institutional clinical pathways, using automated screening tools, collaborating between professionals treating the patient, and designing quality indicators that incorporate functional assessment as a care parameter [85].

## 7 Ethical Considerations

Caring for frail cancer patients frequently raises ethical issues. Should treatment efficacy or quality of life be prioritized? How can autonomy be ensured in cases of cognitive impairment? Is it permissible to withhold treatment if the risk outweighs the benefit? The principle of beneficence requires adapting interventions to the patient's functional status, and the principle of non‐maleficence requires avoiding futile treatments. Respect for autonomy implies shared decision‐making, and justice demands equitable access to specialized care. Cancer patients need to feel involved in decisions about treatments that will prolong their lives [86].

## 8 Conclusions

Frailty is a growing challenge that requires a reorientation of the traditional cancer patient care model. Rather than being an absolute contraindication to treatment, frailty should be understood as a critical clinical parameter that enables the personalization of therapeutic strategies based on individual risk/benefit. This approach promotes more humane and effective medicine. Incorporating comprehensive geriatric assessment and adopting a multidisciplinary approach from the beginning of the diagnostic‐therapeutic process is necessary for achieving this goal. This approach allows for the anticipation of toxicities, the prevention of complications, and the improvement of quality of life. These improvements translate into better clinical and functional outcomes for older patients with cancer.

**FIGURE 1:**
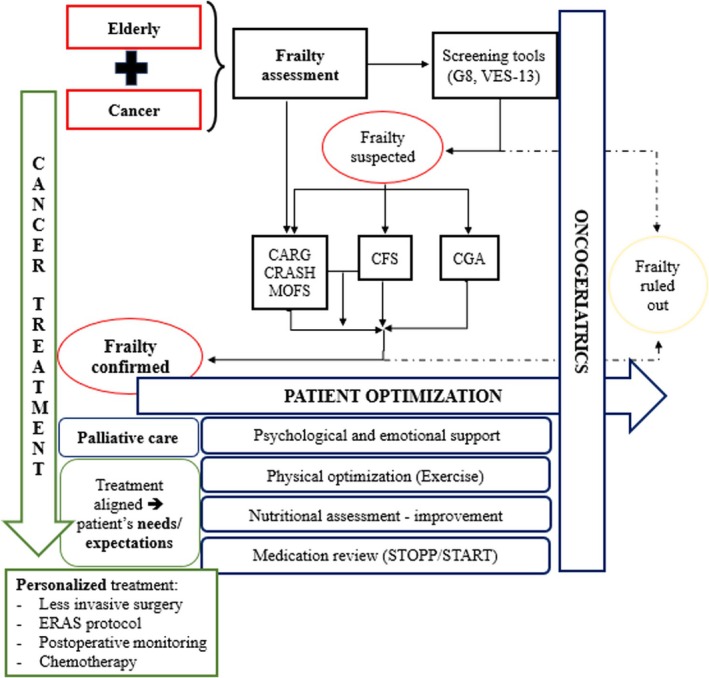
Key interventions to optimize management of frail cancer patients. CARG: cancer and aging research group score; CFS: Clinical Frailty Scale; CGA: Comprehensive Geriatric Assessment; CRASH: Chemotherapy Risk Assessment Scale for High‐Age Patients; ERAS: Enhanced Recovery After Surgery; MOFS: Multidimensional Oncological Frailty Scale; START: Screening Tool to Alert Doctors to Right Treatments; STOPP: Screening Tool of Older Persons' Potentially Inappropriate Prescriptions; VES‐13: Vulnerable Elderly Survey.

Interventions aimed at mitigating frailty, such as nutritional optimization, physical exercise, pharmacological review, and psychosocial support, have proven to be feasible, cost‐effective, and clinically relevant. Implementing oncogeriatric units has emerged as a key structural strategy for achieving these objectives. Additionally, it is essential that healthcare systems integrate frailty as a decision‐making variable in oncology. This integration should include specific training for professionals, the development of institutional protocols, and the promotion of translational and clinical research. Figure 1 summarizes the key findings from the narrative review. For frail cancer patients, it is especially important to focus treatment on the whole patient in a holistic approach, not treating just the disease.

## Author Contributions

**Yanira Hernández‐Aguiar:** formal analysis (equal), investigation (lead), methodology (lead), writing – original draft (equal). **Ángel Becerra‐Bolaños:** conceptualization (lead), data curation (equal), formal analysis (equal), writing – original draft (lead), writing – review and editing (lead). **Aurelio Rodríguez‐Pérez:** funding acquisition (lead), project administration (equal), resources (equal), software (equal), supervision (equal), visualization (supporting), writing – review and editing (equal).

## Funding

The authors have nothing to report.

## Conflicts of Interest

The authors declare no conflicts of interest.

## Data Availability

The present article precludes the sharing of data, as no datasets were generated or analysed during the current study.

## References

[1] A. B. Mitnitski , J. E. Graham , A. J. Mogilner , and K. Rockwood , “Frailty, Fitness and Late‐Life Mortality in Relation to Chronological and Biological Age,” BMC Geriatrics 2 (2002): 1, 10.1186/1471-2318-2-1. 11897015 PMC88955

[2] C. Handforth , A. Clegg , C. Young , et al., “The Prevalence and Outcomes of Frailty in Older Cancer Patients: A Systematic Review,” Annals of Oncology 26, no. 6 (2015): 1091–1101, 10.1093/annonc/mdu540. 25403592

[3] X. Jin , Y. Ren , L. Shao , et al., “Prevalence of Frailty and Prediction of Mortality in Chinese Cancer Patients Using a Frailty Index‐Based Clinical Algorithm—A Multicentre Study,” Cancer Medicine 10, no. 18 (2021): 6207–6217, 10.1002/cam4.4155. 34318626 PMC8446570

[4] K. Komici , L. Bencivenga , N. Navani , et al., “Frailty in Patients With Lung Cancer: A Systematic Review and Meta‐Analysis,” Chest 162, no. 2 (2022): 485–497, 10.1016/j.chest.2022.02.027. 35217002

[5] J. Ripollés‐Melchor , A. Abad‐Motos , and A. Zorrilla‐Vaca , “Enhanced Recovery After Surgery (ERAS) in Surgical Oncology,” Current Oncology Reports 24, no. 9 (2022): 1177–1187, 10.1007/s11912-022-01282-4. 35403970

[6] C. Baethge , S. Goldbeck‐Wood , and S. Mertens , “SANRA—A Scale for the Quality Assessment of Narrative Review Articles,” Research Integrity and Peer Review 4 (2019): 5, 10.1186/s41073-019-0064-8. 30962953 PMC6434870

[7] P. Sharma , K. Zargar‐Shoshtari , J. T. Caracciolo , et al., “Sarcopenia as a Predictor of Overall Survival After Cytoreductive Nephrectomy for Metastatic Renal Cell Carcinoma,” Urologic Oncology 33, no. 8 (2015): 17–23, 10.1016/j.urolonc.2015.01.011. 26094169

[8] L. B. M. Weerink , B. L. van Leeuwen , T. C. Kwee , C. J. C. Lamoth , B. C. van Munster , and G. H. de Bock , “Co‐Occurrence of CT‐Based Radiological Sarcopenia and Frailty Are Related to Impaired Survival in Surgical Oncology,” British Journal of Radiology 98, no. 1168 (2025): 607–613, 10.1093/bjr/tqaf023. 39921891 PMC11919072

[9] C. G. Ethun , M. A. Bilen , A. B. Jani , S. K. Maithel , K. Ogan , and V. A. Master , “Frailty and Cancer: Implications for Oncology Surgery, Medical Oncology, and Radiation Oncology,” CA: A Cancer Journal for Clinicians 67, no. 5 (2017): 362–377, 10.3322/caac.21406. 28731537

[10] D. Weiskopf , B. Weinberger , and B. Grubeck‐Loebenstein , “The Aging of the Immune System,” Transplant International 22, no. 11 (2009): 1041–1050, 10.1111/j.1432-2277.2009.00927.x. 19624493

[11] H. Hipólito‐Reis , J. Santos , P. Almeida , et al., “Implementation of an Oncogeriatric Unit for Frail Older Patients With Breast Cancer: Preliminary Results,” Current Oncology 31, no. 12 (2024): 7809–7819, 10.3390/curroncol31120575. 39727698 PMC11674924

[12] S. Doublet , A. Pagès , Z. A. Thomas , et al., “Systemic Treatment Among Frail Older Patients With Cancer: An Observational Cohort,” Journal of Geriatric Oncology 16, no. 2 (2025): 102177, 10.1016/j.jgo.2024.102177. 39708400

[13] K. James , Y. Jamil , M. Kumar , et al., “Frailty and Cardiovascular Health,” Journal of the American Heart Association 13, no. 15 (2024): e031736, 10.1161/JAHA.123.031736. 39056350 PMC11964060

[14] E. K. Phillips , Y. Huang , E. Regan , et al., “Frailty Associates With Respiratory Exacerbations and Mortality in the COPDGene Cohort,” Aging (Albany NY) 17, no. 7 (2025): 1590–1623, 10.18632/aging.206275. 40622758 PMC12339021

[15] H. Nishikawa , A. Asai , S. Fukunishi , S. Nishiguchi , and K. Higuchi , “Metabolic Syndrome and Sarcopenia,” Nutrients 13, no. 10 (2021): 3519, 10.3390/nu13103519. 34684520 PMC8541622

[16] O. Salehi , I. Zhao , J. Abi Chebl , et al., “Characteristics of Older Patients Undergoing Major Oncological Surgery: Insights From the Geriatric Surgery Verification Program,” Journal of Geriatric Oncololgy 16, no. 2 (2025): 102189, 10.1016/j.jgo.2025.102189. 39818018

[17] T. Sugimoto , H. Arai , and T. Sakurai , “An Update on Cognitive Frailty: Its Definition, Impact, Associated Factors and Underlying Mechanisms, and Interventions,” Geriatrics & Gerontology International 22, no. 2 (2022): 99–109, 10.1111/ggi.14322. 34882939

[18] S. Wang , N. El Jurdi , B. Thyagarajan , A. Prizment , and A. H. Blaes , “Accelerated Aging in Cancer Survivors: Cellular Senescence, Frailty, and Possible Opportunities for Interventions,” International Journal of Molecular Sciences 25, no. 6 (2024): 3319, 10.3390/ijms25063319. 38542292 PMC10970400

[19] F. Seymour , J. Carmichael , C. Taylor , C. Parrish , and G. Cook , “Immune Senescence in Multiple Myeloma—A Role for Mitochondrial Dysfunction?,” Leukemia 36, no. 10 (2022): 2368–2373, 10.1038/s41375-022-01653-7. 35879358

[20] G. A. Abel and H. D. Klepin , “Frailty and the Management of Hematologic Malignancies,” Blood 131, no. 5 (2018): 515–524, 10.1182/blood-2017-09-746420. 29141942

[21] Y. Hernández‐Aguiar , Á. Becerra‐Bolaños , and A. Rodríguez‐Pérez , “Preoperative Diagnosis of Frailty,” Journal of International Medical Research 52, no. 5 (2024): 3000605241251705, 10.1177/03000605241251705. 38818532 PMC11143825

[22] R. Campi , A. Berni , D. Amparore , et al., “Impact of Frailty on Perioperative and Oncologic Outcomes in Patients Undergoing Surgery or Ablation for Renal Cancer: A Systematic Review,” Minerva Urologica e Nefrologica = the Italian Journal of Urology and Nephrology 74, no. 2 (2022): 146–160, 10.23736/S2724-6051.21.04583-3. 34714036

[23] A. Palumbo , S. Bringhen , M. V. Mateos , et al., “Geriatric Assessment Predicts Survival and Toxicities in Elderly Myeloma Patients: An International Myeloma Working Group Report,” Blood 125, no. 13 (2015): 2068–2074, 10.1182/blood-2014-12-615187. 25628469 PMC4375104

[24] M. Hartog , S. J. E. Beishuizen , R. Togo , et al., “Comprehensive Geriatric Assessment, Treatment Decisions, and Outcomes in Older Patients Eligible for Pancreatic Surgery,” Journal of Surgical Oncology 130, no. 8 (2024): 1643–1653, 10.1002/jso.27862. 39290062 PMC11849714

[25] N. Mottet , M. J. Ribal , H. Boyle , et al., “Management of Bladder Cancer in Older Patients: Position Paper of a SIOG Task Force,” Journal of Geriatric Oncololgy 11, no. 7 (2020): 1043–1053, 10.1016/j.jgo.2020.02.001. 32057720

[26] R. Kanesvaran , O. Le Saux , R. Motzer , et al., “Elderly Patients With Metastatic Renal Cell Carcinoma: Position Paper From the International Society of Geriatric Oncology,” Lancet Oncology 19, no. 6 (2018): e317–e326, 10.1016/S1470-2045(18)30125-6. 29893263

[27] J. Klingenschmid , A. Krigers , D. Pinggera , J. Kerschbaumer , C. Thomé , and C. F. Freyschlag , “The Clinical Frailty Scale as Predictor of Overall Survival After Resection of High‐Grade Glioma,” Journal of Neuro‐Oncology 158, no. 1 (2022): 15–22, 10.1007/s11060-022-04001-y. 35467234 PMC9166827

[28] J. Welford , R. Rafferty , K. Hunt , et al., “The Clinical Frailty Scale Can Indicate Prognosis and Care Requirements on Discharge in Oncology and Haemato‐Oncology Inpatients: A Cohort Study,” European Journal of Cancer Care 31, no. 6 (2022): e13752, 10.1111/ecc.13752. 36286099 PMC9788118

[29] R. Iacobescu , L. Boiculese , T. Lunguleac , C. Grigorescu , and S. Antoniu , “Preoperative Frailty as the Strongest Predictor of Postoperative Adverse Events Burden in Patients With Operable Non‐Small Cell Lung Cancer: A Retrospective Analysis,” Expert Review of Respiratory Medicine 19, no. 5 (2025): 475–481, 10.1080/17476348.2025.2487152. 40163011

[30] N. Funamizu , S. Mori , A. Sakamoto , et al., “Novel Modified Frailty Index Predicts Completion of Adjuvant Chemotherapy in Resectable Pancreatic Cancer in a Dual Center Study,” Scientific Reports 15, no. 1 (2025): 17000, 10.1038/s41598-025-02365-5. 40379785 PMC12084558

[31] A. Hurria , K. Togawa , S. G. Mohile , et al., “Predicting Chemotherapy Toxicity in Older Adults With Cancer: A Prospective Multicenter Study,” Journal of Clinical Oncology 29, no. 25 (2011): 3457–3465, 10.1200/JCO.2011.34.7625. 21810685 PMC3624700

[32] M. Extermann , I. Boler , R. R. Reich , et al., “Predicting the Risk of Chemotherapy Toxicity in Older Patients: The Chemotherapy Risk Assessment Scale for High‐Age Patients (CRASH) Score,” Cancer 118, no. 13 (2012): 3377–3386, 10.1002/cncr.26646. 22072065

[33] R. Franchi , C. Okoye , R. Antognoli , et al., “Multidimensional Oncological Frailty Scale (MOFS): A New Quick‐To‐Use Tool for Detecting Frailty and Stratifying Risk in Older Patients With Cancer‐Development and Validation Pilot Study,” Cancers (Basel) 15, no. 5 (2023): 1553, 10.3390/cancers15051553. 36900343 PMC10001199

[34] D. Fusco , A. Ferrini , G. Pasqualetti , et al., “Comprehensive Geriatric Assessment in Older Adults With Cancer: Recommendations by the Italian Society of Geriatrics and Gerontology (SIGG),” European Journal of Clinical Investigation 51, no. 1 (2021): e13347, 10.1111/eci.13347. 32648990

[35] M. Pergolotti , A. M. Deal , G. R. Williams , et al., “Older Adults With Cancer: A Randomized Controlled Trial of Occupational and Physical Therapy,” Journal of the American Geriatrics Society 67, no. 5 (2019): 953–960, 10.1111/jgs.15930. 31034594 PMC6494097

[36] L. M. Kuiper , H. A. Polinder‐Bos , D. Bizzarri , et al., “Epigenetic and Metabolomic Biomarkers for Biological Age: A Comparative Analysis of Mortality and Frailty Risk,” Journals of Gerontology. Series A, Biological Sciences and Medical Sciences 78, no. 10 (2023): 1753–1762, 10.1093/gerona/glad137. 37303208 PMC10562890

[37] F. Liu , T. R. Austin , J. A. Schrack , et al., “Late‐Life Plasma Proteins Associated With Prevalent and Incident Frailty: A Proteomic Analysis,” Aging Cell 22, no. 11 (2023): e13975, 10.1111/acel.13975. 37697678 PMC10652348

[38] J. M. Blodgett , M. U. Pérez‐Zepeda , J. Godin , et al., “Frailty Indices Based on Self‐Report, Blood‐Based Biomarkers and Examination‐Based Data in the Canadian Longitudinal Study on Aging,” Age and Ageing 51, no. 5 (2022): afac075, 10.1093/ageing/afac075. 35524747 PMC9078045

[39] A. Mailliez , A. Guilbaud , F. Puisieux , L. Dauchet , and É. Boulanger , “Circulating Biomarkers Characterizing Physical Frailty: CRP, Hemoglobin, Albumin, 25OHD and Free Testosterone as Best Biomarkers. Results of a Meta‐Analysis,” Experimental Gerontology 139 (2020): 111014, 10.1016/j.exger.2020.111014. 32599147

[40] N. P. Nguyen , U. Karlsson , E. Oboite , et al., “Older Breast Cancer Under Treatment: Unconscious Bias to Undertreat‐Potential Role for the International Geriatric Radiotherapy Group?,” Translational Cancer Research 9, no. Suppl 1 (2020): S228–S235, 10.21037/tcr.2019.10.36. 35117966 PMC8798381

[41] Y. Jauhary , M. R. Gannon , D. Dodwell , et al., “Addressing Frailty in Patients With Breast Cancer: A Review of the Literature,” European Journal of Surgical Oncology 46, no. 1 (2020): 24–32, 10.1016/j.ejso.2019.08.011. 31439357

[42] A. Farcet , L. de Decker , V. Pauly , et al., “Frailty Markers and Treatment Decisions in Patients Seen in Oncogeriatric Clinics: Results From the ASRO Pilot Study,” PLoS One 11, no. 2 (2016): e0149732, 10.1371/journal.pone.0149732. 26918947 PMC4769181

[43] G. Ugolini , F. Ghignone , D. Zattoni , G. Veronese , and I. Montroni , “Personalized Surgical Management of Colorectal Cancer in Elderly Population,” World Journal of Gastroenterology 20, no. 14 (2014): 3762–3777, 10.3748/wjg.v20.i14.3762. 24833841 PMC3983435

[44] H. J. Boyle , S. Alibhai , L. Decoster , et al., “Updated Recommendations of the International Society of Geriatric Oncology on Prostate Cancer Management in Older Patients,” European Journal of Cancer 116 (2019): 116–136, 10.1016/j.ejca.2019.04.031. 31195356

[45] A. Q. Akinoso‐Imran , M. O'Rorke , F. Kee , H. Jordao , G. Walls , and F. J. Bannon , “Surgical Under‐Treatment of Older Adult Patients With Cancer: A Systematic Review and Meta‐Analysis,” Journal of Geriatric Oncololgy 13, no. 4 (2022): 398–409, 10.1016/j.jgo.2021.11.004. 34776385

[46] A. Becerra‐Bolaños , Y. Hernández‐Aguiar , and A. Rodríguez‐Pérez , “Preoperative Frailty and Postoperative Complications After Non‐Cardiac Surgery: A Systematic Review,” Journal of International Medical Research 52, no. 9 (2024): 3000605241274553, 10.1177/03000605241274223. 39268763 PMC11406619

[47] C. DuMontier , W. Dale , A. C. Revette , et al., “Ethics of Overtreatment and Undertreatment in Older Adults With Cancer,” BMC Medical Ethics 26, no. 1 (2025): 105, 10.1186/s12910-025-01255-9. 40707902 PMC12291383

[48] T. Wang , J. Jiang , Z. Song , et al., “Prevalence of Frailty and Its Predictors Among Patients With Cancer at the Chemotherapy Stage: Systematic Review,” JMIR Cancer 11 (2025): e69936, 10.2196/69936. 40706088 PMC12289293

[49] E. D. Duchesneau , D. H. Kim , T. Stürmer , et al., “Frailty Trajectories Following Adjuvant Chemotherapy and Mortality in Older Women With Breast Cancer,” JAMA Network Open 8, no. 3 (2025): e250614, 10.1001/jamanetworkopen.2025.0614. 40072432 PMC11904708

[50] S. Pranikoff , V. L. Ayer Miller , H. Heiling , et al., “Frail Young Adult Cancer Survivors Experience Poor Health‐Related Quality of Life,” Cancer 128, no. 12 (2022): 2375–2383, 10.1002/cncr.34196. 35319782 PMC9133201

[51] A. Delaney , C. R. Howell , K. R. Krull , et al., “Progression of Frailty in Survivors of Childhood Cancer: A St. Jude Lifetime Cohort Report,” Journal of the National Cancer Institute 113, no. 10 (2021): 1415–1421, 10.1093/jnci/djab033. 33720359 PMC8633430

[52] E. Dotan , L. C. Walter , I. S. Browner , et al., “NCCN Guidelines Insights: Older Adult Oncology, Version 1.2021,” Journal of the National Comprehensive Cancer Network 19, no. 9 (2021): 1006–1019, 10.6004/jnccn.2021.0043. 34551388

[53] J. K. Dhesi , N. P. Lees , and J. S. Partridge , “Frailty in the Perioperative Setting,” Clinical Medicine 19, no. 6 (2019): 485–489, 10.7861/clinmed.2019-0283. 31732590 PMC6899239

[54] J. Dourado , A. Wolf , M. Herrera Rodriguez , et al., “ERAS Protocol in Colorectal Surgery Is Effective in Octogenarians: A Retrospective Cohort Study,” Surgery Open Science 24 (2025): 86–91, 10.1016/j.sopen.2025.03.004. 40166625 PMC11957660

[55] J. Chen , C. Hong , R. Chen , M. Zhou , and S. Lin , “Prognostic Impact of a 3‐Week Multimodal Prehabilitation Program on Frail Elderly Patients Undergoing Elective Gastric Cancer Surgery: A Randomized Trial,” BMC Gastroenterology 24, no. 1 (2024): 403, 10.1186/s12876-024-03490-7. 39528916 PMC11556218

[56] F. M. Runzer‐Colmenares , D. Urrunaga‐Pastor , M. A. Roca‐Moscoso , J. De Noriega , O. Rosas‐Carrasco , and J. F. Parodi , “Frailty and Vulnerability as Predictors of Chemotherapy Toxicity in Older Adults: A Longitudinal Study in Peru,” Journal of Nutrition, Health & Aging 24, no. 9 (2020): 966–972, 10.1007/s12603-020-1404-6. PMC12280679 33155622

[57] L. Balducci , “Aging, Frailty, and Chemotherapy,” Cancer Control 14, no. 1 (2007): 7–12, 10.1177/107327480701400102. 17242666

[58] S. L. Crowder , A. I. Hoogland , B. J. Small , et al., “Associations Among Frailty and Quality of Life in Older Patients With Cancer Treated With Chemotherapy,” Journal of Geriatric Oncololgy 13, no. 8 (2022): 1149–1155, 10.1016/j.jgo.2022.08.010. PMC9871794 36008271

[59] K. Bylow , S. G. Mohile , W. M. Stadler , and W. Dale , “Does Androgen‐Deprivation Therapy Accelerate the Development of Frailty in Older Men With Prostate Cancer?: A Conceptual Review,” Cancer 110, no. 12 (2007): 2604–2613, 10.1002/cncr.23084. 17960609

[60] J. T. Wu , J. Corrigan , C. Su , et al., “The Performance Status Gap in Immunotherapy for Frail Patients With Advanced Non‐Small Cell Lung Cancer,” Cancer Immunology, Immunotherapy 73, no. 9 (2024): 172, 10.1007/s00262-024-03763-w. 38954019 PMC11219626

[61] T. Stratulat Alexa , I. Alexa , and S. Antoniu , “Palliative Immunotherapy in the Frail Elderly: Non‐Small Cell Lung Cancer,” BMJ Supportive & Palliative Care 12, no. 2 (2022): 191–193, 10.1136/bmjspcare-2021-003223. 34728473

[62] Z. Güzelöz and U. Gök Balci , “The Impact of Radiotherapy on Frailty Patients Aged 65 and Over,” Cureus 15, no. 10 (2023): e46351, 10.7759/cureus.46351. 37790869 PMC10544825

[63] N. P. Nguyen , L. Kim , J. Thariat , et al., “Immunotherapy and Modern Radiotherapy Technique for Older Patients With Locally Advanced Head and Neck Cancer: A Proposed Paradigm by the International Geriatric Radiotherapy Group,” Cancers (Basel) 14, no. 21 (2022): 5285, 10.3390/cancers14215285. 36358703 PMC9654379

[64] R. B. Parikh , R. A. Kirch , T. J. Smith , and J. S. Temel , “Early Specialty Palliative Care—Translating Data in Oncology Into Practice,” New England Journal of Medicine 369, no. 24 (2013): 2347–2351, 10.1056/NEJMsb1305469. 24328469 PMC3991113

[65] J. Apóstolo , R. Cooke , E. Bobrowicz‐Campos , et al., “Effectiveness of Interventions to Prevent Pre‐Frailty and Frailty Progression in Older Adults: A Systematic Review,” JBI Database of Systematic Reviews and Implementation Reports 16, no. 1 (2018): 140–232, 10.11124/JBISRIR-2017-003761. 29324562 PMC5771690

[66] S. C. Hayes , R. U. Newton , R. R. Spence , and D. A. Galvao , “The Exercise and Sports Science Australia Position Statement: Exercise Medicine in Cancer Management,” Journal of Science and Medicine in Sport 22, no. 11 (2019): 1175–1199, 10.1016/j.jsams.2019.05.003. 31277921

[67] S. I. Mishra , R. W. Scherer , C. Snyder , P. M. Geigle , D. R. Berlanstein , and O. Topaloglu , “Exercise Interventions on Health‐Related Quality of Life for People With Cancer During Active Treatment,” Cochrane Database of Systematic Reviews 2012, no. 8 (2012): CD008465, 10.1002/14651858.CD008465.pub2. 22895974 PMC7389071

[68] K. S. Courneya , R. J. Segal , J. R. Mackey , et al., “Effects of Aerobic and Resistance Exercise in Breast Cancer Patients Receiving Adjuvant Chemotherapy: A Multicenter Randomized Controlled Trial,” Journal of Clinical Oncology 25, no. 28 (2007): 4396–4404, 10.1200/JCO.2006.08.2024. 17785708

[69] K. Barnes , E. Hladkowicz , K. Dorrance , et al., “Barriers and Facilitators to Participation in Exercise Prehabilitation Before Cancer Surgery for Older Adults With Frailty: A Qualitative Study,” BMC Geriatrics 23, no. 1 (2023): 356, 10.1186/s12877-023-03990-3. 37280523 PMC10242997

[70] J. Arends , V. Baracos , H. Bertz , et al., “ESPEN Expert Group Recommendations for Action Against Cancer‐Related Malnutrition,” Clinical Nutrition 36, no. 5 (2017): 1187–1196, 10.1016/j.clnu.2017.06.017. 28689670

[71] N. Kiss , J. Loeliger , M. Findlay , et al., “Clinical Oncology Society of Australia: Position Statement on Cancer‐Related Malnutrition and Sarcopenia,” Nutrition and Dietetics 77, no. 4 (2020): 416–425, 10.1111/1747-0080.12631. 32803904 PMC7540290

[72] J. P. Turner , S. Shakib , and J. S. Bell , “Is My Older Cancer Patient on Too Many Medications?,” Journal of Geriatric Oncololgy 8, no. 2 (2017): 77–81, 10.1016/j.jgo.2016.10.003. 27840102

[73] M. Carollo , V. Boccardi , S. Crisafulli , et al., “Medication Review and Deprescribing in Different Healthcare Settings: A Position Statement From an Italian Scientific Consortium,” Aging Clinical and Experimental Research 36, no. 1 (2024): 63, 10.1007/s40520-023-02679-2. 38459218 PMC10923734

[74] P. Esper and D. Heidrich , “Symptom Clusters in Advanced Illness,” Seminars in Oncology Nursing 21, no. 1 (2005): 20–28, 10.1053/j.soncn.2004.10.004. 15807053

[75] L. Grassi , R. Caruso , M. B. Riba , et al., “Anxiety and Depression in Adult Cancer Patients: ESMO Clinical Practice Guideline,” ESMO Open 8, no. 2 (2023): 101155, 10.1016/j.esmoop.2023.101155. 37087199 PMC10163167

[76] M. G. Deng , F. Liu , Y. Liang , K. Wang , J. Q. Nie , and J. Liu , “Association Between Frailty and Depression: A Bidirectional Mendelian Randomization Study,” Science Advances 9, no. 38 (2023): eadi3902, 10.1126/sciadv.adi3902. 37729413 PMC10511184

[77] M. A. Mafla‐España and O. Cauli , “Non‐Pharmacological Interventions for Managing the Symptoms of Depression in Women With Breast Cancer: A Literature Review of Clinical Trials,” Diseases 13, no. 3 (2025): 80, 10.3390/diseases13030080. 40136619 PMC11941554

[78] J. Lehmann , D. Riedl , A. Nickels , et al., “Associations of Age and Sex With the Efficacy of Inpatient Cancer Rehabilitation: Results From a Longitudinal Observational Study Using Electronic Patient‐Reported Outcomes,” Cancers (Basel) 15, no. 6 (2023): 1637, 10.3390/cancers15061637. 36980523 PMC10046728

[79] A. Guerin , Z. Ap Thomas , C. Nagera‐Lazarovici , et al., “Comprehensive Geriatric Assessment and Early Treatment Failure in Nonagenarian Patients With Cancer, a Retrospective Monocentric Study,” Cancer Epidemiology 97 (2025): 102830, 10.1016/j.canep.2025.102830. 40288114

[80] B. L. van Leeuwen , S. R. Kristjansson , and R. A. Audisio , “Should Specialized Oncogeriatric Surgeons Operate Older Unfit Cancer Patients?,” European Journal of Surgical Oncology 36, no. 1 (2010): S18–S22, 10.1016/j.ejso.2010.06.018. 20591605

[81] L. André , G. Antherieu , A. Boinet , et al., “Oncological Treatment‐Related Fatigue in Oncogeriatrics: A Scoping Review,” Cancers (Basel) 14, no. 10 (2022): 2470, 10.3390/cancers14102470. 35626074 PMC9139887

[82] G. Singh , L. Morant , M. Bedra , et al., “Value of a Multidisciplinary Geriatric Oncology Committee on Patient Care in a Community‐Based, Academic Cancer Center,” Journal of Geriatric Oncololgy 15, no. 4 (2024): 101771, 10.1016/j.jgo.2024.101771. 38615579

[83] G. L. Banna , O. Cantale , M. M. Haydock , et al., “International Survey on Frailty Assessment in Patients With Cancer,” Oncologist 27, no. 10 (2022): e796–e803, 10.1093/oncolo/oyac133. 35905085 PMC9526491

[84] V. Goede , “Frailty and Cancer: Current Perspectives on Assessment and Monitoring,” Clinical Interventions in Aging 18 (2023): 505–521, 10.2147/CIA.S365494. 37013130 PMC10066705

[85] C. Mac Eochagain , A. Barrell , V. Slavova‐Boneva , et al., “Implementation of a Geriatric Oncology Service at the Royal Marsden Hospital,” Journal of Geriatric Oncololgy 15, no. 2 (2024): 101698, 10.1016/j.jgo.2023.101698. 38219333

[86] L. D. Tuesen , H. H. Bülow , A. S. Ågård , S. M. Strøm , E. Fromme , and H. I. Jensen , “Patient‐Physician Conversations About Life‐Sustaining Treatment: Treatment Preferences and Participant Assessments,” Palliative & Supportive Care 21, no. 1 (2023): 20–26, 10.1017/S1478951521001875. 36814149

